# A Novel Training and Collaboration Integrated Framework for Human–Agent Teleoperation

**DOI:** 10.3390/s21248341

**Published:** 2021-12-14

**Authors:** Zebin Huang, Ziwei Wang, Weibang Bai, Yanpei Huang, Lichao Sun, Bo Xiao, Eric M. Yeatman

**Affiliations:** 1 Department of Bioengineering, Imperial College London, London SW7 2BX, UK; zebin.huang20@imperial.ac.uk (Z.H.); ziwei.wang@imperial.ac.uk (Z.W.); 2 Department of Computing, Imperial College London, London SW7 2BX, UK; wbbai@imperial.ac.uk (W.B.); b.xiao@imperial.ac.uk (B.X.); 3 School of Education, Communication & Society, King’s College London, London SE5 9RJ, UK; lichao.sun@kcl.ac.uk; 4 Department of Electrical and Electronic Engineering, Imperial College London, London SW7 2BX, UK; e.yeatman@imperial.ac.uk

**Keywords:** human–agent interaction, teleoperation, reinforcement learning

## Abstract

Human operators have the trend of increasing physical and mental workloads when performing teleoperation tasks in uncertain and dynamic environments. In addition, their performances are influenced by subjective factors, potentially leading to operational errors or task failure. Although agent-based methods offer a promising solution to the above problems, the human experience and intelligence are necessary for teleoperation scenarios. In this paper, a truncated quantile critics reinforcement learning-based integrated framework is proposed for human–agent teleoperation that encompasses training, assessment and agent-based arbitration. The proposed framework allows for an expert training agent, a bilateral training and cooperation process to realize the co-optimization of agent and human. It can provide efficient and quantifiable training feedback. Experiments have been conducted to train subjects with the developed algorithm. The performances of human–human and human–agent cooperation modes are also compared. The results have shown that subjects can complete the tasks of reaching and picking and placing with the assistance of an agent in a shorter operational time, with a higher success rate and less workload than human–human cooperation.

## 1. Introduction

Teleoperation helps perform long-distance interaction tasks and thus ensures operation safety. A network-based communication channel isolates a human operator from a potentially hazard interaction environment, which is therefore regarded as the special case of cyber-physical systems. In this regard, teleoperation triggers the systemic revolution of human-in-the-loop operation [1], providing a universal platform to medical diagnosis [2] and fault diagnosis [3,4,5] for industrial applications, which has been widely applied to space robotics [6,7,8,9], medical surgery [10,11,12,13] and deep-sea exploration [14,15].

Regarding complex teleoperation tasks, the traditional single-master/single-slave (SM/SS) mode cannot meet the increased requirements of robustness and flexibility. Therefore, introducing another operator becomes a possible solution to extend teleoperation applications [16,17]. In contrast to SM/SS mode, multi-master/single-slave (MM/SS) teleoperation can be applied to online training and multiple manipulator collaboration, where shared commands at master side are integrated and sent to the slave robot via weight distribution. The reliability and effectiveness can be enhanced by MM/SS teleoperation, not only enabling fine operation but also reducing operation error through collaborative decision making [18,19,20].

Despite these advantages of MM/SS systems, human factors might introduce stability issues to the operation process. When faced with unstructured environments and unexpected accidents, human operators can be mentally stressed, leading to subjective cognition bias and flawed decisions. To solve this problem, we can introduce reinforcement learning (RL) to the human-in-the-loop system. RL is a popular approach for policy optimization that has seen various applications in the robotics domain [17,21,22,23]. Complex and accurate high-speed calculations are easy for agents, which can implement policy optimization under certain rule constraints. Most RL works focus on autonomous [24,25,26,27] and multiple-agent tasks [28,29,30,31] that do not exhibit human traits. Despite the promise of high-precision maneuvering by agents, human guidance is still needed for specific teleoperation tasks. It is difficult for robots to perceive unstructured environments accurately, which might result in unsafe behavior during the learning process [32].

In addition, there is a lack of systems for training humans to adapt to cooperative patterns with agents. This poses a challenge to existing MM/SS teleoperation systems: how to train the agent which adapts to human operation and then design a training and cooperation system based on the trained agent. Although some RL works show agents can be trained by setting the reward function, they only focused on the unilateral training process and did not propose whether the the agent’s policy was based on rule constraints [28,29,30,33,34]. The human–agent cooperative mechanism compared with human–human was not taken into account either. Moreover, some existing works modeled human actions as part of the RL environment [26,35,36]. Nevertheless, humans are characterized by dynamic real-time interaction rather than the passive environment in terms of human-oriented teleoperation.

In this paper, a truncated quantile critics (TQC) RL-based training and collaboration integrated framework is proposed for human–agent teleoperation that encompasses assessment and agent-based arbitration. This framework describes the learning, training and cooperation relationships among experts, agents and novices based on different experience levels. Different from training agents, we focus on agent-to-human training and bilateral cooperation between a human and agent, in which the agent trains novices and trained novices work with the agent.

## 2. Methods

**Figure 1:**
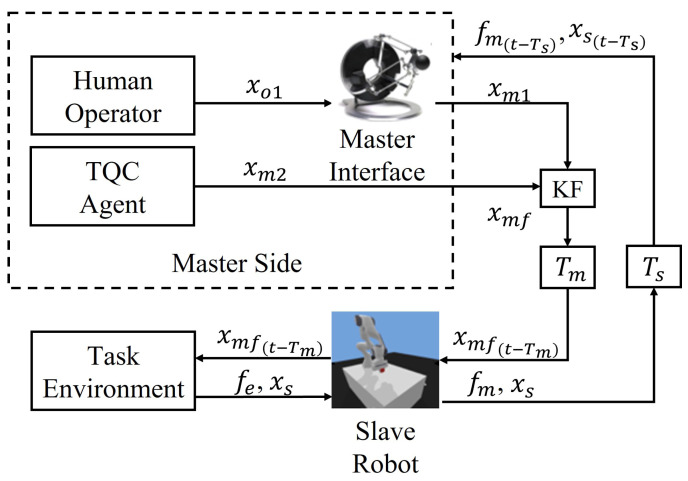
Overview of the human–agent cooperative teleoperation framework based on RL and KF.

The proposed cooperation system is mainly composed of a master interface, TQC agent and the slave robot interacting with environments. The master commands from the operator and agent will be fused through a Kalman filter (KF) before being sent to the slave side, where $x_{m1}$ and $x_{m2}$ represent the commands defined in Cartesian space from the human operator and agent, respectively. The interactive force $f_{e}$ will also feedback to the master side via a communication channel, which results in the latency $T_{m}$ and $T_{s}$ in the closed-loop system. $f_{m}$ is the force mapped to the master interface. The arguments of the variables involved in the diagram are omitted for simplicity, as shown in Figure 1. It is worth pointing out that the proposed framework also allows for human–human cooperation, which can be realized through replacing the agent with a human.

**Figure 2:**
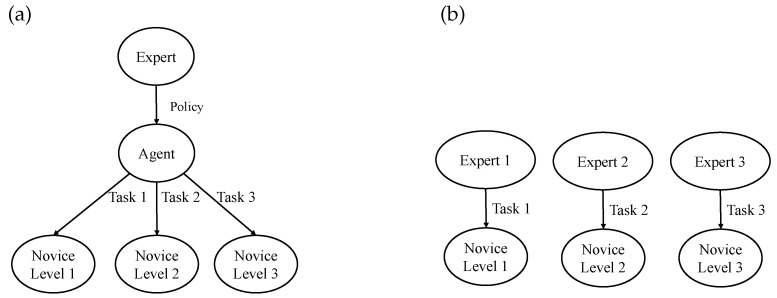
Comparison of training methods based on RL and one-to-one training mode. (**a**) RL-based training mode. (**b**) Traditional one-to-one training mode.

Before performing the human–agent cooperation, a critical issue is how to improve agent and operator skills to achieve a similar level of operation for both. Compared with the traditional one-to-one training mode [37,38] (see Figure 2b), the agent-based training method [39] is employed in this paper, which is able to provide one-to-many training for the novices at different levels, as demonstrated in Figure 2a. The adopted agent can effectively implement the expert’s constraint rules to provide standardized actions and quantitative feedback. Moreover, the agent-based training approach significantly reduces the need for expert resources and enables simultaneous remote training of different novices.

**Figure 3:**
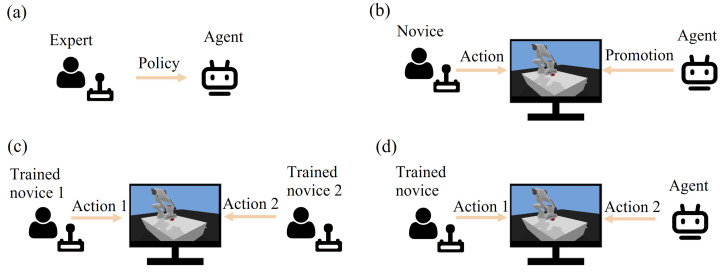
Training and collaboration procedure. (**a**) Expert trains agent. (**b**) Agent trains novice. (**c**) Human cooperates with human. (**d**) Agent cooperates with human.

The overall training and collaboration procedure is depicted in Figure 3. In order to render the training and cooperation framework, the first step is to formulate certain constraints through expert experience and train the agent with specific operational tasks through RL, as shown in Figure 3a. Then, with the aid of the trained agent, a training and cooperation system will be accessible for novices (Figure 3b). The developed system is beneficial for the training and cooperation of novices, including human–human (Figure 3c) and human–agent (Figure 3d) cooperation modes. The dominance factors of the agent and human will change dynamically based on their performance and task constraints. When the performance gap between operator and expert is large, the agent will play a relatively dominant role, guiding the novice user; as the performance gap narrows, the agent’s dominance will gradually shift to the user side.

### 2.1. Expert Trains Agent

Robotic manipulation tasks are characterized by high dimensions and continuous state space, including kinematic information on the robot and environment. When attempting to solve such problems, a dense reward function is difficult to define and use to guide the robot through the learning process, leading to a high time cost. To overcome this issue, we introduce hindsight experience replay (HER) [40] and the TQC method to train the agent. In this way, the agent is trained through the reward function, speed and position constraints based on expert rules.

#### 2.1.1. Hindsight Experience Replay

With the HER algorithm, the robot can learn from the high-dimension continuous state-action space, which facilitates increasing the reward density for the agent. Prior to completing the learning of a good strategy, the agent could complete the current RL for any state experienced in the sequence. If these states are regarded as task goals, the agent can obtain a large number of positive rewards, thereby promoting the learning process.

The HER algorithm additionally defines the task target space *G* under the basic RL framework, in which each target element g∈G corresponds to a reward mapping $r_{g}:S\times A\to R$. At the beginning of each episode, by sampling the initial state in the distribution $p\left(s_{0},g\right)$ and the task goal, the task goal remains unchanged during the phase of interaction with the environment. In each timestep, the agent’s strategy input includes both the current state and current task goal, namely π:S×G→A, and obtains an instant reward $R_{t}=r_{g}\left(s_{t},a_{t}\right)$.

The input of the *Q* function includes state, action and goal, namely $Q^{\pi }\left(s_{t},a_{t},g\right)=E\left[\sum_{i=t}^{\infty }\gamma^{i}R_{i}\right]$ with γ∈(0,1) being the discount factor. For the multi-goal RL task, assume that the target element *g* corresponds to a mapping $f_{g}:S\to \left\{0,1\right\}$, that is, the goal of the agent is to reach the state *s* by interacting with the environment, so as to satisfy $f_{g}\left(s\right)=1$. The target can also be specified to satisfy certain attributes in the state. For example, if the state is used to describe the two-dimensional coordinates of the horizontal plane, where the agent is currently located in $S=R^{2}$. The task goal is to render the current state satisfying the given horizontal coordinates $g=x_{g}$, then, there exists $f_{g}\left(\left(x,y\right)\right)=\left[x=g|g\in G=R\right]$. In addition, we can define the target mapping description. For any given state *s*, there exists a mapping such that $m:S\to G,s.t.\forall_{s\in S}f_{m(s)}\left(s\right)=1$.

#### 2.1.2. Truncated Quantile Critics

We train the agent with the TQC algorithm [41] for two experimental applications, namely reaching and picking and placing (P&P) tasks. The state, action and reward for this experiment are defined as follows.

1. The actual state of the robot: angles and angular velocities of the robot joints, the poses of the objects and their linear and angular velocities.
2. The robot observation state: in the reaching task, the observation space contains the position and velocity of the end-effector. In the P&P task, the observation space contains the position and velocity of the end-effector and the objects’ pose.
3. The initial state and target distribution: in both tasks, the end-effector starts from a fixed position in each round. The robot end-effector is 20 cm above the surface. The initial position of the object is randomly sampled from the surface within a square. If the initial state already meets the task target, the initial state and target will be resampled.
4. Task goal: the target position *g* that the robot needs to move the object to within a certain error range $\varepsilon_{R}$. Thus, the goal is accomplished with $f_{g}\left(s\right)=1$ and $\left[d\left(s_{obj},g\right)\leq \varepsilon_{R}\right]=1$, where $s_{obj}$ is the position of the target object, and the output $d:R^{3}\times R^{3}\to R$ is the Euclidean distance between the two inputs.
5. The reward function is defined as the negative bool value if the distance between the achieved and target positions is lower than the threshold.

### 2.2. Agent Trains Novice

**Figure 4:**
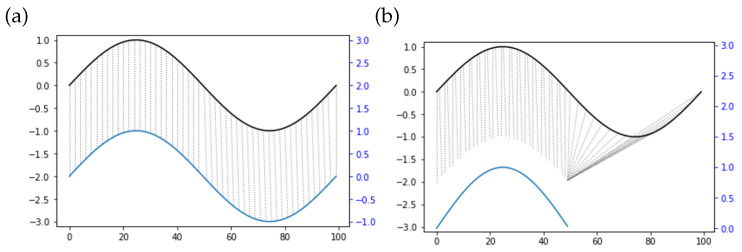
Comparison of points matching and feature matching. The abscissa is the time series sequence, and the ordinate is the time series value. (**a**) Points matching. (**b**) Feature matching.

Similarity assessment is used to evaluate the training result of the operator. We check the similarity of operations from an expert agent and operator, and then train the operator through improving the similarity level. In teleoperation, similarity is reflected by the time delay and the operating speed difference when human operators conduct the same trajectory. Operators may behave at different speeds to perform the same trajectory. Apart from that, the displacement can only occur on the time axis in terms of different time series. With reverted displacement as an example, the two time series present the identity. Based on the above complicated cases, it is difficult to successfully measure the similarity between two time series with the application of point-to-point matching methods, such as Euclidean distance (Figure 4a). The dynamic time warping (DTW) algorithm [42] is therefore used to match time series with different lengths through prolonging and restricting the time series. In accordance with Figure 4b, the top and bottom solid lines denote the two time series, and the dashed lines between them stand for the similar points. Since a discrete-time sequence characterizes the motion trajectory collected, the DTW algorithm can avoid unrecognizable problems due to different lengths. The similarity of two trajectories could be therefore calculated as follows. 

Suppose the trajectory outputs by the agent and operator are $T_{a}$ and $T_{o}$, namely $T_{a}=\left\{T_{a1},T_{a2},\ldots ,T_{aN}\right\}\in R^{2\times N}$ and $T_{o}=\left\{T_{o1},T_{o2},\ldots ,T_{oM}\right\}\in R^{2\times M}$. We consider the warp path as $W=w_{1},w_{2},\ldots ,w_{k},\ldots ,w_{K},\max\left\{M,N\right\}\leq K\leq M+N$. The distance of the warp path follows the cost matrix D, whose element is described by

(1) $$D\left(i,j\right)=d_{ij}+\min\left\{D\left(i-1,j\right),D\left(i,j-1\right),D\left(i-1,j-1\right)\right\}$$

where D(M,N) is the minimum distance of warp path *W*, which is regarded as a metric to evaluate the similarity. $d_{ij}≜\mathrm{Dist}\left(T_{ai},T_{oj}\right)$ is the Euclidean distance between the two data points. The two trajectory sequences are therefore matched through the DTW algorithm. The smaller the D(M,N) we calculate, the more similar the two trajectory sequences are.

### 2.3. Agent Cooperates with Human

The KF module is used to fuse the two commands (i.e., $x_{m1}$ and $x_{m2}$) and output the fused command $x_{mf}$. A discrete control process can be described as

(2) X(k)=AX(k−1)+ω(k)

(3) Z(k)=HX(k)+V(k)

where $X\left(k\right)≜x_{mf}\left(k\right)$ is the system state which contains position and velocity signals at the *k*th sample. *H* is the observation matrix. *A* is the state-transition matrix. $Z\left(k\right)≜{\left[x_{m1}\left(k\right),x_{m2}\left(k\right)\right]}^{T}$ is the observation state. ω(k) and V(k) represent process and measurement noise, respectively. They are assumed to be white Gaussian noise, and their covariances are *Q* and *R*, respectively. Based on the previously collected data, a one-step state estimate can be deduced from the system process

(4) $$\begin{array}{c} X(k|k-1)=AX(k-1|k-1)\\ P\left(k|k-1\right)=AP\left(k-1|k-1\right)A^{T}+Q \end{array}$$

where X(k|k−1) is the state prediction and X(k−1|k−1) the optimal result based on the previous state. P(k|k−1) and P(k−1|k−1) are the covariances of X(k|k−1) and X(k−1|k−1), respectively. Based on the observed value at the *k*th sample and the estimated value at the k−1th sample, we can obtain

(5) $$\begin{array}{c} X(k|k)=X(k|k-1)+K(k)(Z(k)-HX(k|k-1))\\ K\left(k\right)=P\left(k|k-1\right)H^{T}{\left(HP\left(k|k-1\right)H^{T}+R\right)}^{-1}\\ P(k|k)=(I-G(k-1))P(k|k-1) \end{array}$$

where K(k) is the Kalman gain, G(k)=K(k)H and P(k|k) is the error covariance matrix. With the block matrix $G\left(k\right)=[G{\left(k\right)}_{\left(1\right)},G{\left(k\right)}_{\left(2\right)}]$, we can derive from (5) that

(6) $$x_{mf}\left(k|k\right)=\left(I-G\left(k\right)\right)x_{mf}\left(k|k-1\right)+G{\left(k\right)}_{\left(1\right)}x_{m1}\left(k\right)+G{\left(k\right)}_{\left(2\right)}x_{m2}\left(k\right).$$

## 3. Experiment

We have conducted a series of experiments with ten healthy subjects (9 male, age 21 ± 3, 9 right-handed) without motor impairment. None of the subjects had experience with haptic devices. The experiment was approved by the Research Ethics Committee of Imperial College London (No. ICREC-18IC4816). Each subject was informed about the experiment and signed a consent form before the test.

### 3.1. Experimental Platform

**Figure 5:**
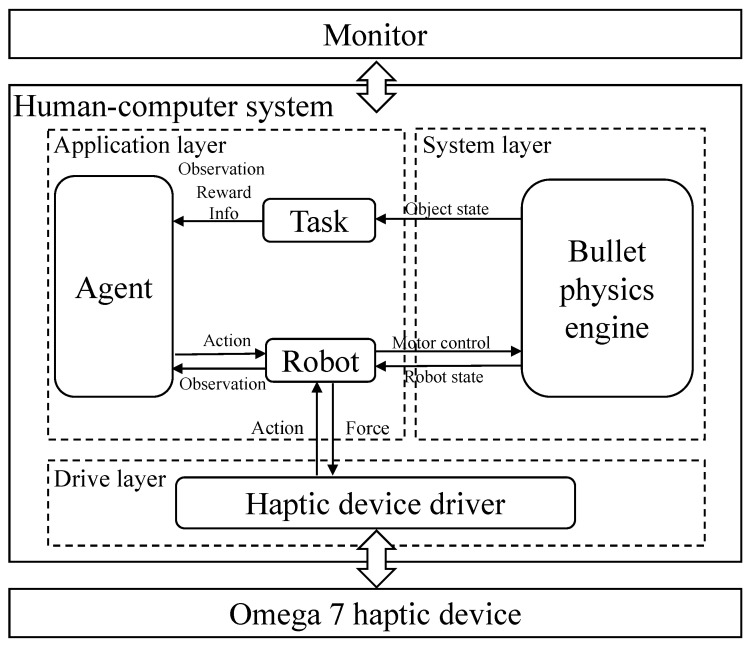
Simulation system architecture diagram.

The experiment was conducted based on the simulation platform. The system architecture diagram is shown in Figure 5, which consists of three main components: a human–computer simulation system, a visual display and a haptic interface. The haptic device (Omega.7, Force Dimension) was used to collect six degree-of-freedom (DoF) motion information and provide three-DoF force feedback in translations. The subject held the handle of the haptic device to remotely control the slave robot in Cartesian space. The human–computer system rendered the corresponding visual and haptic feedback information to the operator through a monitor and haptic device.

**Figure 6:**
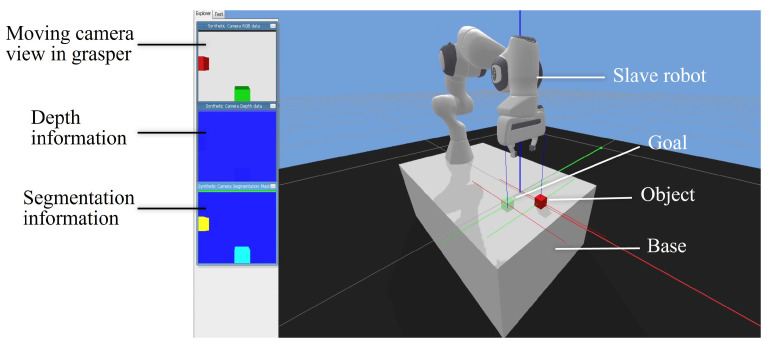
GUI for the teleoperation system. The interface was divided into a display area and an operation area. The three windows in the display area (**left** panel) presented the visual feedback of gripper’s camera in top view, depth information and segmentation information, respectively. The operation area (**right** panel) showed the operation robot in perspective view under a global camera.

The software components of the system can be divided into driver layer, system layer and application layer. The driver layer processed haptic feedback information and drove the haptic device. In the system layer, the Bullet physics engine [43] was employed to support Pybullet for the simulation. The application layer included modules of the agent, task and robot. The simulation platform used the Franka Panda robot (Franka Emika GmbH Inc., Munich, Germany) as the slave robot to interact with the environment. The visual display is shown in Figure 6 and this graphic user interface (GUI) was designed based on Pybullet and Stable-Baselines3 [44]. The main view for the operator was the slave robot under a global camera (Figure 6, right panel). There were also three auxiliary windows on the left side of the GUI to provide user 3D information. The first view was extracted from the moving camera in the gripper of the robot, providing dynamic interaction information. The second window could provide depth information of the objects, and the third segmentation view could reflect the profiles of the objects.

### 3.2. Experimental Procedure

**Figure 7:**
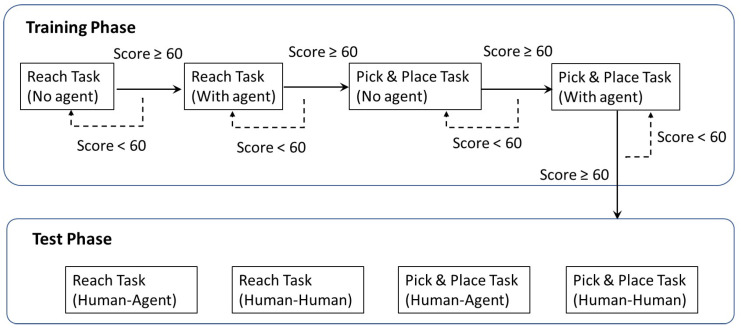
Experiment procedure overview.

The whole experiment included training and test phases (Figure 7). The training session was designed to allow the subjects to become familiar with the teleoperation and reach the entry level of the task operation, where the agent would intermittently provide guidance trajectory for the novice in this phase. Then, in the test phase, we investigated human–human (HH) and human–agent (HA) cooperation to further check whether the agent could help the operator perform the task as a human operator. Finally, an assessment phase was conducted to collect the participants’ subjective responses to HH and HA cooperation modes.

#### 3.2.1. Tasks

The reaching and P&P tasks were designed to reflect the common actions in teleoperation. The robot was set to move in a workspace of 30 × 30 × 20 cm${}^{3}$. The targets were shown within the robot’s workspace.

**Figure 8:**
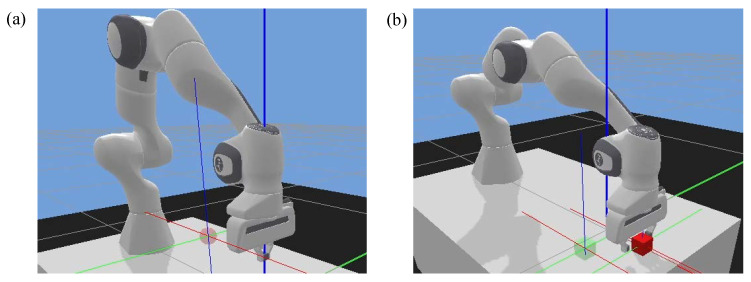
Two task scenarios. (**a**) Reaching task. (**b**) P&P task.

1. Reaching task (Figure 8a): this task was developed to test the subject’s motor control capability. The robot gripper remained closed in this task. A red target ball was randomly generated on the table. The subject was required to control the slave robot through the hand controller to reach the target as fast and accurately as possible. If the subject realized the goal within the update time (13 s), a new target would be shown.
2. P&P task (Figure 8b): this task tested both the subject’s movement and grasping control abilities, which required a high level of coordination. In this task, a random red cube was generated on the table plane. The subject needed to control the robot end-effector to pick up the cube and place it in the target position (green transparent cube). The successful grasping should satisfy both position and force criteria, i.e., the gripper moves to the target and lifts it up using proper force. This grasping force was calculated by the physical engine of the robot controller through checking the deformation of the object surface. A new target would be shown if the goal was achieved or update time was reached. The update time for this task was 30 s.

#### 3.2.2. Training Phase

In this phase, we provided the novices with visual cues and an operation score to learn the operation efficiently. For each task, there were two modes for training, namely without or with an agent. It should be noted that the agent in the training phase represents providing a guidance trajectory instead of involving control commands. At the end of each session, a performance score (0 to 100) was shown to the subject. It was derived from the similarity between the actual motion and guidance trajectory, where the latter represents the optimal operation in which an agent learned from an expert. If the subject’s performance score reaches 60, it is supposed that he/she has gained the control skills required for the test phase. Otherwise, the subject needs to continue a second training session for the corresponding mode. The criterion of the score and time limits was set based on trial and error using data of the reference value taken from the experimenters.

#### 3.2.3. Test Phase

**Figure 9:**
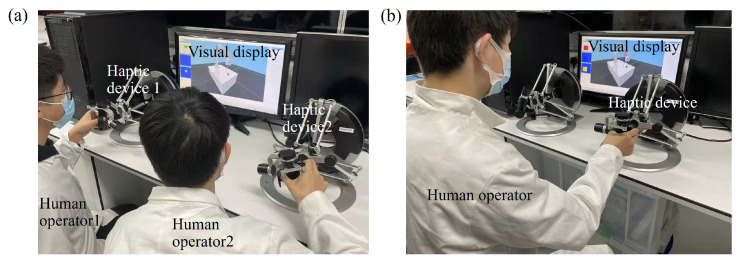
Experiment setup in (**a**) human–human cooperation mode and (**b**) human–agent cooperation mode.

There was no guided trajectory shown on the screen in this phase. Subjects were involved in two different modes of cooperation, HH and HA cooperation tasks. The subjects were also told whether they would cooperate with a human or agent before each mode. In the HH cooperation mode, ten subjects were randomly paired into five groups to conduct the cooperation task. In the HA mode, one subject cooperated with the agent to perform the same task. At the end of the experiment, all subjects were subjectively assessed by questionnaires. Data such as operational time and task success rates were recorded for all trials. The experimental scene is shown in Figure 9.

### 3.3. Evaluation Measures

The performance of the subjects was evaluated through both quantitative measures and subjective measures.

#### 3.3.1. Quantitative Measures

1. Success rate: the success rate is the percentage of successfully completed trials to the total number of trials. The task is defined as successfully completed when the object is reached (i.e., the distance between robot end-effector and the center of the target cube is less than 0.05 m) within 13 s in the reaching task. In the P&P task, successful operations include reaching, grasping the object and moving it to the target position within 30 s. Except satisfying the position criterion, the grasping forces of the gripper should be large enough to lift the object.
2. Operation time: the operation time is the time from the display of the target to the successful arrival or placement. In the failed trial, the operation time is the update time.

#### 3.3.2. Subjective Measures

The subjects were invited to fill in the questionnaire after test phases (Table 1), where Q1 describes the comparison with human operation without the assistance of an agent or other subject. The questionnaire was designed to evaluate their subjective responses in mental effort, temporal demand, agency, performance, task difficulty and robot/human assistance. All questionnaire items were constructed using a 5-point Likert scale.

## 4. Results

In this section, we will show the experimental results of test phase in both performance metrics and questionnaire responses. A Shapiro–Wilk test was conducted to examine the data distribution. Both quantitative and subjective measurements were not normally distributed. Thus, we used non-parametric Wilcoxon signed rank test to check the differences of human–human and human–agent control modes.

### 4.1. Performance Measures

**Figure 10:**
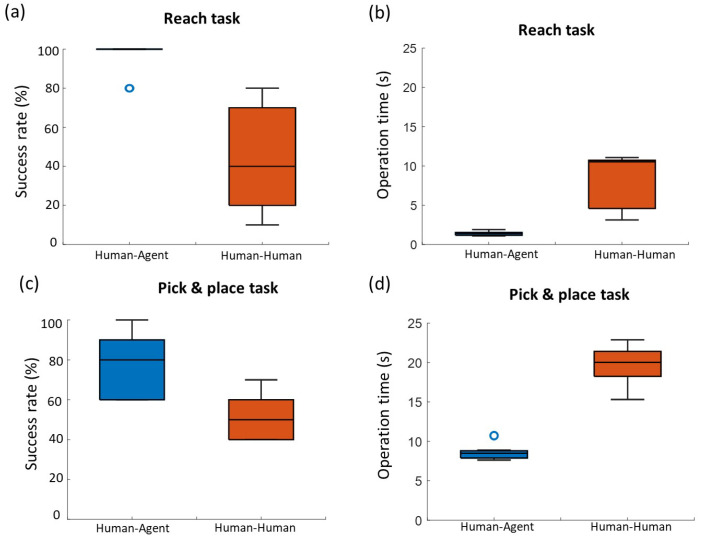
Subjects’ performance result of reaching task in (**a**) success rate and (**b**) operation time; and the P&P task in (**c**) success rate and (**d**) operation time.

Figure 10a shows the result of the success rate for the reaching task. The average success rate of human–human cooperation mode was 44.0% with 27.2% deviation. In contrast, the average success rate of human–human cooperation mode arrived at 96.0% with lower deviation of 8.4%. The addition of robotic agent significantly improves the success rate compared with a human partner (*p* = 0.007). Similarly, the assistance of an agent is also reflected in the operation time. The subjects took 8.0 ± 3.5 s in average to complete the task in human–human cooperation, while human–agent saved more than 80% of the time than human–human mode, thereby significantly improving the operation efficiency (*p* = 0.005). The agent played an important role in human–agent cooperation with average weights from 0.74 to 0.95.

Compared to the reaching task, the P&P task required relatively more time to be performed (see Figure 10d). The average success rate for human–agent cooperation in the P&P task was about 77.0% lower than that in the reaching task. On the other hand, the success rate for human–human cooperation in the P&P task was not affected by the task complexity and even a little higher than that in the reaching task. This result suggests that the current assistance strategies of the robotic agent are more suitable for simple motion tasks. However, the advantages of human–agent cooperation in both metrics were still obvious compared with the human–human cooperation (Figure 10c,d). The average operation time for human–human cooperation was 19.5 ± 2.8 s, while the human and agent spent about 8.6 ± 0.9 s (*p* = 0.005). The success rate of the human–agent mode was significantly higher than human–human mode (*p* = 0.007). In the process of the P&P task, the average weights for agent in human–agent cooperation were within the range of [0.647,0.929].

### 4.2. Subjective Assessment

**Figure 11:**
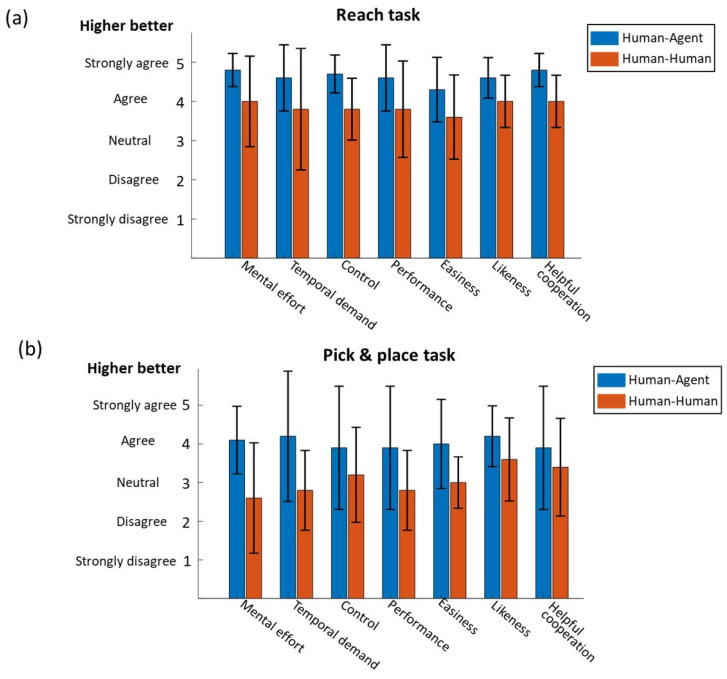
Questionnaire result for (**a**) reach task and (**b**) pick & place task.

The questionnaire result for the reaching task is shown in Figure 11a. In general, the subjects’ responses were positive on the reaching task. The average response ranges for human–human and human–agent cooperation were [3.6,4.0] and [4.3,4.8], respectively, which were both above the neutral score. The subjects felt that cooperating with the agent required less mental effort compared to that with a human partner with a marginally significant difference (*p* = 0.05). This might be due to the fact that the agent could provide efficient and consistent assistance to the operator and the subject could trust the agent and follow its guidance. Then, it is reasonable that operating with an agent was easier in terms of the subjects’ feelings (*p* = 0.02). In addition, the subjects believed they were able to control the robot with the agent better (*p* = 0.02). This indicates that although the agent may contribute more to the task completion, the operator still had the autonomy with even stronger feelings of control. However, cooperating with the agent and human partner did not cause different feelings on temporal demand and performance (*p* = 0.08, 0.1, respectively). However, from subjective perspectives, the subjects preferred cooperating with an agent than a human partner (*p* = 0.01), and they believed the robotic agent was more helpful to perform the task than cooperating with a human partner (*p* = 0.02).

The subjective responses for the P&P task are shown in Figure 11b. As the P&P task consists not only of reaching, but also grasping, moving and loading, it is obvious that the subjective scores of the P&P task (Figure 11b) were lower than those of the reaching task (Figure 11a) in all metrics. The average responses were from 2.6 to 3.6 for human–human mode and 3.9 to 4.2 for human–agent mode. Similar to the reaching task, the subjects felt human–agent cooperation was easier than human–human (*p* = 0.008) in the P&P task. Although the subjects felt the task was easier with an agent, they were uncertain whether the agent partner or human partner helped them more. There is no significant difference in assistance from the human and agent partner (*p* = 0.3). Furthermore, subjects felt their performance was better when cooperating with an agent (3.9 ± 1.6) but there was no significant difference between the human or agent partner (*p* = 0.06). In addition, different from the responses in the reaching task, subjects did not feel an obvious difference in control (*p* = 0.08) or preference (*p* = 0.1) when cooperating with an agent or human. However, it is worth noting that human–agent cooperation required significantly less mental effort (*p* = 0.02) and temporal demand (*p* = 0.03) than human–human cooperation in the P&P task.

## 5. Discussion

The experimental results have validated the proposed cooperative framework for multi-master/single-slave teleoperation and proved that human–agent cooperation is superior to human–human cooperation. With the help of a Kalman filter, the human and agent commands can be balanced and fused by time-varying weights. Compared with the previous telerobotic schemes [8,9], the introduced TQC agent extends the traditional single-master/single-slave teleoperation paradigm to overcome subjective operation errors, which enables the collaborative agent to assist on the master side. Instead of correcting the master command by introducing a co-pilot on the slave side [17], the master commands are blended on the master side, which overcomes the uncertainty caused by the co-pilot and reduces the computational burden for the slave side.

In general, it is found that cooperating with an agent could reduce the subjects’ mental effort and make the task operation easier compared to cooperating with a human partner in both the reaching and P&P task. Compared to a human partner, the proposed agent plays a fixed role as an assistant, where the degree of assistance is determined dynamically by the subject’s operation performance. In contrast, a human partner is unpredictable, so a lack of understanding of each other’s proficiency would result in complicated interactive behaviors between a human and human, including cooperation, collaboration and competition [45]. The human–human interactive behaviors could switch among these three taxonomies, so it is difficult to achieve the interaction tasks in the absence of informative communication. In addition, the subjects felt the agent was more helpful and they had better control than with a human partner in the reaching task. Since the P&P task consists of multiple sub-processes (i.e., reaching, grasping, moving and loading), more conflicted behaviors might be triggered. As a result, the subjective assessment gap in performing the P&P task is more pronounced than that in the reaching task.

In the experiment, the agent played a relatively dominant role in both tasks with the average dynamic weights greater than 0.647. On the one hand, this result indicates that the designed agent could effectively assist a human operator to reduce their workload and mental load. On the other hand, the human operator trusted the cooperated agent and was inclined to cede some of the autonomy to the agent in terms of tasks. The agent in the reaching task tended to be assigned more weights than in the complex P&P task, due to the fact that subjects were possibly more confident in the agent performing simple movements. Regarding the P&P task, the agent partner was still superior to the human partner but its assistance to the operator was affected by the complexity of the task. Correspondingly, the operator was determined to take back some control and assign relatively less weight to the agent. However, it should be noticed that, in the current experiment setting, the novice operators were trained in a relatively short time and their capability may still be at the entry level, which explained the high weights in both tasks from another point of view.

## 6. Conclusions

The teleoperation control framework for human–agent collaboration is investigated in this paper. A reinforcement learning algorithm and Kalman filter in the proposed cooperative framework allowed for novice training and human–agent collaboration simultaneously. The TQC agent is beneficial to improve the training compared with traditional one-expert-to-one-novice training mode. The experimental results have quantitatively shown that the proposed framework can improve the success rate and operation time for the reaching task and P&P task with the collaboration of the designed agent. The questionnaire result also sheds light on the fact that the collaborating agent partner facilitates reducing human mental loads.

Although the proposed human–agent partnership effectively improves the operation efficiency and reduces human mental efforts, there are some aspects we could further improve in the proposed framework. Firstly, the assistance role of the agent is initially assumed to be fixed. However, a flexible role of the agent is more practical for physical human–robot interaction. We will integrate the human intent prediction into the system which enables real-time estimation of the operator’s state and adjusts the role of the assistant agent accordingly. Secondly, the proposed agent is suitable for simple reaching tasks but less adaptive to complicated P&P tasks. Our future research will investigate how a human transfers the authority to his/her partner in more complicated interaction tasks. Based on this, we will develop more natural, supportive and task-specific agent for a human operator or autonomous agent–agent control to perform multiple operation activities.

## Figures and Tables

**Table 1:** Questionnaire for human–human and human–agent control modes.

ID	Questions
Q1	I felt less mental effort.
Q2	I felt I have sufficient time to complete the task.
Q3	I felt I can control the robot well.
Q4	I felt my performance is good.
Q5	I felt the task is easy.
Q6	I liked the task.
Q7	I thought my collaborators are helpful to me.

## Data Availability

The data presented in this study are available on request from the corresponding author. The data are not publicly available due to privacy and ethical.

## Abbreviations

The following abbreviations are used in this manuscript:

KF	Kalman Filter
TQC	Truncated Quantile Critics
RL	Reinforcement Learning
P & P	Pick and Place
HER	Hindsight Experience Replay
HH	Human–Human
HA	Human–Agent
